# All-optical ultrafast polarization switching with nonlinear plasmonic metasurfaces

**DOI:** 10.1126/sciadv.adk3882

**Published:** 2024-02-21

**Authors:** Heng Wang, Zixian Hu, Junhong Deng, Xuecai Zhang, Jiafei Chen, Kingfai Li, Guixin Li

**Affiliations:** ^1^ Department of Materials Science and Engineering, Southern University of Science and Technology, Shenzhen 518055, China.; ^2^ Shenzhen Institute for Quantum Science and Engineering, Southern University of Science and Technology, Shenzhen 518055, China.; ^3^ Institute for Applied Optics and Precision Engineering, Southern University of Science and Technology, Shenzhen 518055, China.

## Abstract

Optical switching has important applications in optical information processing, optical computing, and optical communications. The long-term pursuit of optical switch is to achieve short switching time and large modulation depth. Among various mechanisms, all-optical switching based on Kerr effect represents a promising solution. However, it is usually difficult to compromise both switching time and modulation depth of a Kerr-type optical switch. To circumvent this constraint, symmetry selective polarization switching via second-harmonic generation (SHG) in nonlinear crystals has been attracting scientists’ attention. Here, we demonstrate SHG-based all-optical ultrafast polarization switching by using geometric phase controlled nonlinear plasmonic metasurfaces. A switching time of hundreds of femtoseconds and a modulation depth of 97% were experimentally demonstrated. The function of dual-channel all-optical switching was also demonstrated on a metasurface, which consists of spatially variant meta-atoms. The nonlinear metasurface proposed here represents an important platform for developing all-optical ultrafast switches and would benefit the area of optical information processing.

## Teaser

Nonlinear plasmonic metasurface represents an important platform for all-optical ultrafast polarization switching.

## INTRODUCTION

Optical switches play key roles in the systems that using light as an information carrier, such as optical computing and optical communications. Switching time and modulation depth are the two key parameters of an optical switch. While the former determines the upper limit of the operating frequency, the latter determines the signal-to-noise ratio. Therefore, developing optical switches with both short switching times and large modulation depths is highly desirable in the optics community. The physical mechanisms of conventional optical switches include electro-optical effect (*1*–*4*), acousto-optical effect (*5*), magneto-optical effect (*6*–*8*), and so on. However, such switches usually suffer from the low switching speed. To circumvent this constraint, all-optical switches based on the nonlinear optical processes have been attracting scientists’ attention. In an all-optical switch, the pump beam is usually used to change the properties of the materials, which in turn changes the transmittance or reflectance of the probe beam. The Kerr effect, in which the refractive index of a material depends on the intensity of the pumping light, is one of the most important mechanisms for all-optical switching. In the past years, a variety of designs of all-optical switches based on on-chip interferometer (*9*), waveguide ring resonator (*10*), photonic crystal cavity (*11*–*13*), plasmonic waveguide (*14*), and metasurfaces (*15*–*22*) were developed. In these studies, there are usually trade-offs between switching time and modulation depth of all-optical switches. The large modulation depth of all-optical switches usually requires long light-matter interaction length, which will inevitably increase the switching time (*9*, *10*, *12*–*14*). On the other hand, if the switching time of all-optical switches are short, then the modulation depth are usually small (*17*, *21*–*23*). The modulation depth of the all-optical switches could be increased by applying the materials with large optical nonlinearities, such as lithium niobate (*24*), epsilon-near-zero materials (*23*, *25*–*28*), and two-dimensional (2D) materials (*29*).

Second-harmonic generation process provides an excellent solution for compromising both the switching time and modulation depth of an all-optical switch. Second-harmonic generation (SHG) usually occurs in materials with centrosymmetry breaking, such as metals (*30*), optical crystals (*31*, *32*), 2D materials (*33*, *34*), and metasurfaces (*35*). To achieve a SHG-based optical switch, several mechanisms including hot-electron transfer (*36*), Floquet engineering (*37*), and photocurrent-induced symmetry breaking (*38*) have been proposed. In addition, symmetry selective polarization switching in the SHG (*39*), third-harmonic generation (*40*), and four-wave mixing (*41*) processes were successfully demonstrated on the 2D materials platform, which provides an alternative approach for all-optical switching. In such kind of all-optical switches, the modulation depth is usually very large, and the switching time is only limited by the pulse width of the pumping light. Recent progress of nonlinear plasmonic metasurface offers an alternative approach for developing high-performance optical switch. In this area, by artificially designing the meta-atoms with centrosymmetry breaking in a metasurface, both the phase and polarization of the SH waves (*42*–*44*) and terahertz waves (*45*) could be well controlled.

Here, we demonstrate all-optical ultrafast and large-modulation-depth polarization switching by using the SHG process with a plasmonic metasurface. The metasurface consists of gold meta-atoms with threefold rotational symmetry (*C*_3_), which exhibit strong plasmonic resonance in the near-infrared regime. By varying the time delay of the two pump beams with orthogonal linear polarizations, the polarization of the SH wave experiences an ultrafast switching. A switching time of 521 fs and a near-unity modulation depth were experimentally obtained. We also demonstrated that the plasmonic metasurface allows for dual-channel all-optical switching and could be used for optical information encoding.

## RESULTS

### Working principle of the metasurface optical switch

**Fig. 1.**
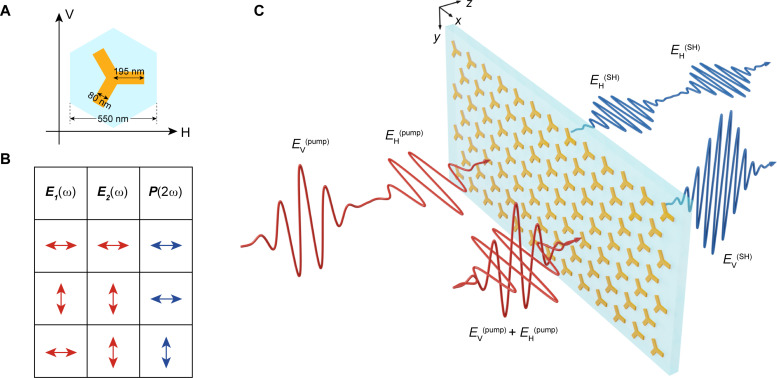
All-optical polarization switching of the SH waves with a plasmonic metasurface. (**A**) The geometrical parameters of a *C*_3h_ gold meta-atom. The arm length, width, and thickness of the meta-atom are 195, 80, and 30 nm, respectively. The meta-atoms are arranged in a hexagonal lattice with a period of 550 nm. (**B**) Different combinations of the two pump beams (red arrow) with H- (horizontal) and V- (vertical) polarization correspond to the generated SH waves (blue arrow) with H- or V-polarization. (**C**) Schematic diagram of all-optical polarization switching of SH waves with the plasmonic metasurface. When the time delay between two pump pulses is larger than the pulse duration, H-polarized SH waves are generated. When two pump pulses coincide in time domain, the V-polarized SH waves can be observed.

A uniform plasmonic metasurface was designed and fabricated for all-optical polarization switching of the SH wave. Each gold meta-atom of the metasurface has threefold rotational symmetry, as shown in Fig. 1A. If one of the three arms is along horizontal (H) direction, then it is denoted as *C*_3h_. As each plasmonic meta-atom itself can be used to control the phase, polarization and amplitude of the generated SH waves, the coupling effect between the neighboring meta-atoms can be greatly reduced by choosing appropriate the lattice distance. The gold meta-atoms are arranged in a hexagonal lattice with a period of 550 nm. The area size of the metasurface is around 500 μm by 500 μm. The plasmonic metasurface radiates SH waves under the ultrafast laser excitation owing to the broken inversion symmetry of the gold meta-atoms. The metasurface was fabricated on a 15-nm-thick indium tin oxide (ITO) film–coated glass substrate. In the gold-ITO hybrid system, both the epsilon-near-zero effect and the inversion symmetry breaking of the *C*_3_ meta-atom play important roles for enhancing the SHG intensity of the device (*46*). When the time delay between the two pump pulses is larger than the pulse duration, H-polarized SH waves are generated. In comparison, when two pump pulses coincide in time domain, V-polarized SH waves can be observed, as shown in Fig. 1 (B and C).

### Nonlinear optical properties of the plasmonic metasurface

**Fig. 2.**
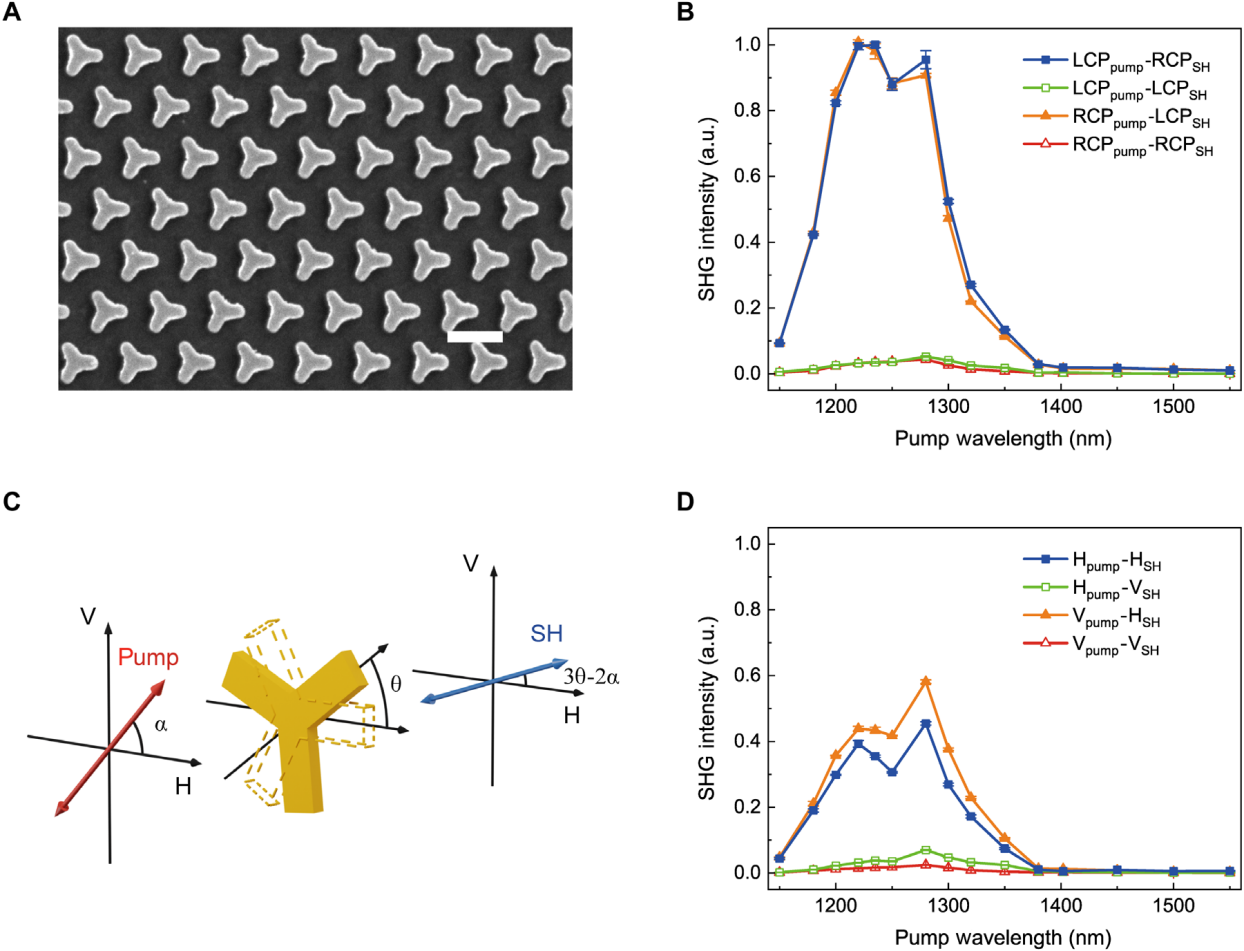
Nonlinear optical properties of the uniform plasmonic metasurface. (**A**) SEM image of the plasmonic metasurface. Scale bar, 500 nm. (**B**) Measured wavelength-dependent SHG intensities for circularly polarized pump wave. Four kinds of circular polarization combinations of the pump waves and SH waves are recorded. (**C**) The polarization angle of the pump (α) and the SH wave (3θ − 2α), where θ is the orientation angle of the *C*_3_ meta-atom. (**D**) Measured wavelength-dependent SHG intensities for linearly polarized pump wave. The results are normalized to the maximum SHG values in (B). H, horizontal polarization; V, vertical polarization; a.u., arbitrary units.

The scanning electron microscopy (SEM) image of the uniform plasmonic metasurface is shown in Fig. 2A. First, the basic nonlinear optical properties of the metasurfaces were characterized by using an optical parametric oscillator (OPO) system (Materials and Methods). This is mainly because the OPO system has low pulse energy and high repetition frequency. Compared to a kilohertz femtosecond laser, the OPO system usually brings less damage to the plasmonic devices to obtain similar counts of SHG signals. As shown in Fig. 2B, the wavelength-dependent SHG intensities for pump waves with circular polarizations were measured. The metasurface has a high SHG efficiency for the pump wavelength between 1180 and 1300 nm. From these results, we choose the pump wavelength of 1220 nm in the following all-optical switching experiment. On the basis of the symmetry selection rule of the SHG process for the *C*_3_ meta-atoms (*43*, *47*), the SH waves with opposite circular polarization to that of the pump wave (LCP_pump_-RCP_SH_ and RCP_pump_-LCP_SH_) are much stronger than that in the case of LCP_pump_-LCP_SH_ and RCP_pump_-RCP_SH_ measurements. According to the theory of nonlinear geometric phase (*47*–*49*), the polarization angle of the SH wave is related to both the polarization angle of the pump wave and the orientation angle of the *C*_3_ meta-atom. The linearly polarized pump wave with polarization angle α with respective to *x* axis could be expressed as [ cos (α)  sin (α)]^T^, and it can be represented as a superposition of left and right circular polarization (LCP and RCP) states $\left[{|L\rangle }_{\omega }\text{exp}\left(i\alpha \right)+{|R\rangle }_{\omega }\text{exp}\left(-i\alpha \right)\right]/\sqrt{2}$ , where $|L\rangle ={\left[1\;i\right]}^{T}/\sqrt{2}$ and $|R\rangle ={\left[1\;-i\right]}^{T}/\sqrt{2}$ denote the LCP and RCP vectors, and the subscript ω and 2ω indicate the angular frequency of the pump waves and SH waves. For the *C*_3_ meta-atom with an orientation angle of θ, the nonlinear geometric phase of the SH wave is −3σθ, where σ = ± 1 represent LCP and RCP state of the pump wave, respectively. The corresponding RCP and LCP states of the SH waves could be represented as ${|R\rangle }_{2\omega }\text{exp}\left(-i3\theta \right)\text{exp}\left(i2\alpha \right)/\sqrt{2}$ and ${|L\rangle }_{2\omega }\text{exp}\left(i3\theta \right)\text{exp}\left(-i2\alpha \right)/\sqrt{2}$ . The synthesized polarization of the SH wave is given by ${\left[\begin{array}{cc} \text{cos}\left(3\theta -2\alpha \right) & \text{sin}\left(3\theta -2\alpha \right) \end{array}\right]}^{T}$ , indicating a polarization angle of 3θ − 2α (Fig. 2C).

The polarization properties of the SH wave can be explained by the second-order susceptibility of a *C*_3_ meta-atom (section S1). For a *C*_3h_ meta-atom (θ = 0°), the nonvanishing elements of the second-order susceptibility tensor are $\chi_{\mathrm{xxx}}^{\left(2\right)}=-\chi_{\mathrm{xyy}}^{\left(2\right)}=-\chi_{\mathrm{yxy}}^{\left(2\right)}=-\chi_{\mathrm{yyx}}^{\left(2\right)}$ . When it is excited by the H-polarized pump wave (α = 0°), only $\chi_{\mathrm{xxx}}^{\left(2\right)}$ needs to be taken into account. Therefore, the polarization direction of the generated SH wave is along H-direction (3θ − 2α = 0°). In comparison, when it is excited by the V-polarized pump wave (α = 90°), tensor element $\chi_{\mathrm{xyy}}^{\left(2\right)}$ is involved in the SHG process, and then the SH wave is also H-polarized (3θ − 2α = 180°). The wavelength-dependent SHG intensities with linear polarization were measured by using the OPO system (Materials and Methods), and the results are shown in Fig. 2D. Both the H- and V-polarized pump waves ( ${|H\rangle }_{\omega }$ and ${|V\rangle }_{\omega }$ ) can be used to independently generate H-polarized SH waves ( ${|H\rangle }_{2\omega }$ and $-{|H\rangle }_{2\omega }$ ), where $|H\rangle ={\left[1\;0\right]}^{T}$ and $|V\rangle ={\left[0\;1\right]}^{T}$ denote the H- and V-polarization vectors, respectively. However, when pump waves with H- and V-polarization coincide in the time domain, the generated SH wave is switched to V-polarization due to the contribution of the last two tensor elements $\chi_{\mathrm{yxy}}^{\left(2\right)}$ and $\chi_{\mathrm{yyx}}^{\left(2\right)}$ . Assuming the electric field amplitudes of the two pump waves are equal, the overall polarization state could be written as ${|H\rangle }_{\omega }+{|V\rangle }_{\omega }$ or $\left(1-i\right){|L\rangle }_{\omega }/\sqrt{2}+\left(1+i\right){|R\rangle }_{\omega }/\sqrt{2}$ . The corresponding RCP and LCP states of the SH waves could be derived as $-i\sqrt{2}{|R\rangle }_{2\omega }$ and $i\sqrt{2}{|L\rangle }_{2\omega }$ . Therefore, the synthesized polarization of the SH wave is $-2{|V\rangle }_{2\omega }$ , which is perpendicular to that of SH wave in the former case and therefore has the potential to be used for all-optical ultrafast polarization switching.

### All-optical ultrafast polarization switching

To demonstrate the function of all-optical ultrafast polarization switching, the uniform plasmonic metasurface is excited by two pulsed pump waves with H- and V-polarization. The two pump beams have same frequency, which can be easily obtained by splitting one beam into two. For the *C*_3h_ meta-atoms, the polarization state of the SH wave could be derived as (section S1)

$$\left[\begin{array}{c} P_{x}^{\left(2\omega \right)}\\ P_{y}^{\left(2\omega \right)} \end{array}\right]\propto \left[\begin{array}{c} A_{1}^{2}\left(t+\tau \right)\text{exp}\left[-2i\omega \left(t+\tau \right)\right]-A_{2}^{2}\left(t\right)\text{exp}\left(-2i\omega t\right)\\ -2A_{1}\left(t+\tau \right)A_{2}\left(t\right)\left[-i\omega \left(2t+\tau \right)\right] \end{array}\right]$$ (1)

where *t* is the time, and $A_{1}\left(t+\tau \right)$ and $A_{2}\left(t\right)$ are the temporal envelope functions of the electric fields of the two pump waves. $A_{1}\left(t+\tau \right)$ is in H-polarization with a variable delay τ, and $A_{2}\left(t\right)$ is in V-polarization with a fixed delay. Assuming the electric field amplitudes of the two pump waves are equal and the pulse shapes in the time domain are same as each other, i.e., $A_{1}=A_{2}=A$ . For a Gaussian pulse, the envelope function of the electric field of the pump wave in time domain could be written as $A\left(t\right)=\text{exp}\left[-t^{2}/\left(2\tau_{0}^{2}\right)\right]$ , where τ_0_ is a parameter that determines the pulse width. Substituting *A*(*t*) in Eq. 1, the τ-dependent averaged SHG power is then derived as

$$\left[\begin{array}{c} I_{x}^{\left(2\omega \right)}\\ I_{y}^{\left(2\omega \right)} \end{array}\right]\propto \left[\begin{array}{c} 1-\text{cos}\left(2\omega \tau \right)\text{exp}\left[-\tau^{2}/\left(2\tau_{0}^{2}\right)\right]\\ 2\text{exp}\left[-\tau^{2}/\left(2\tau_{0}^{2}\right)\right] \end{array}\right]$$ (2)

**Fig. 3.**
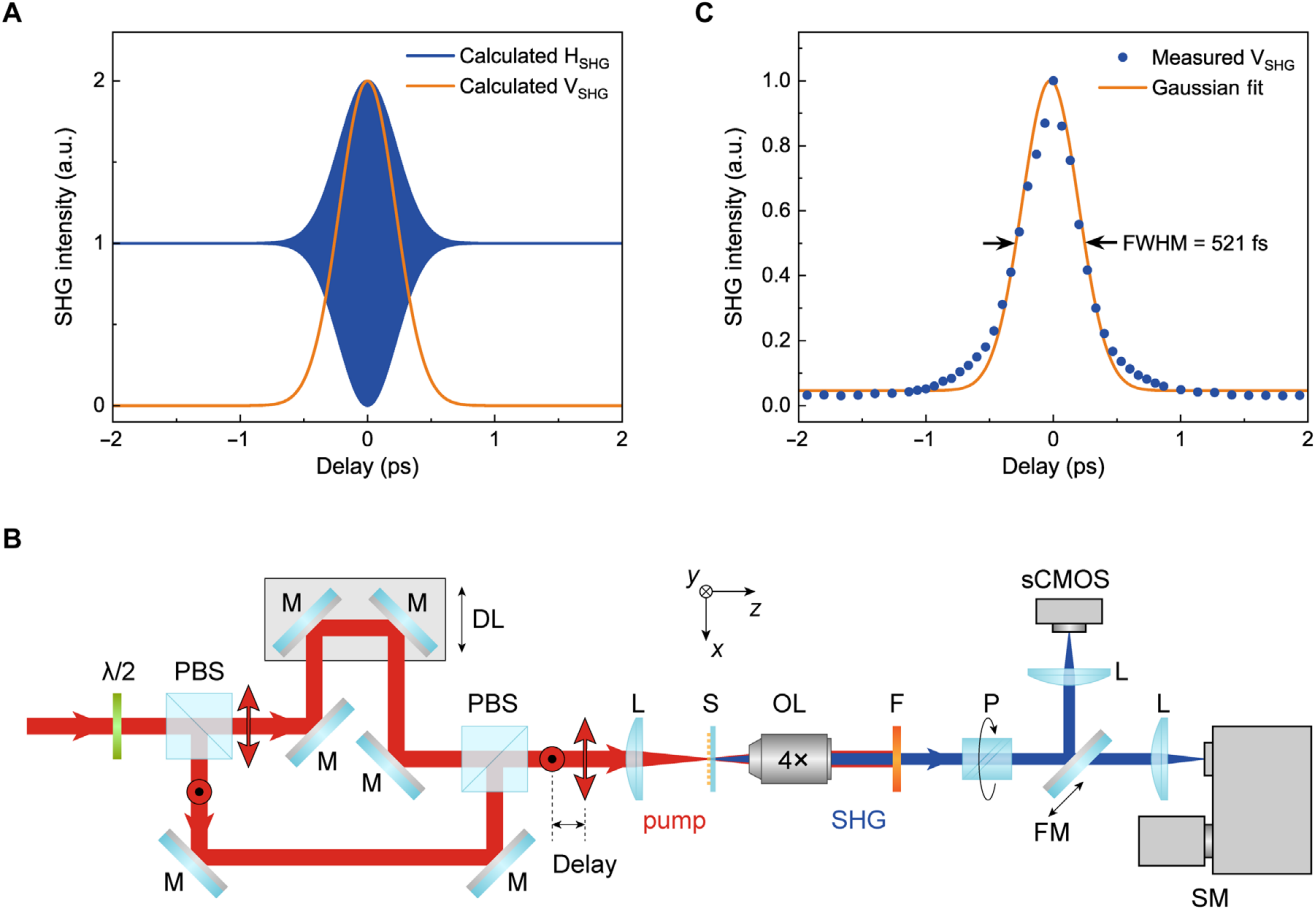
All-optical ultrafast polarization switching with the uniform plasmonic metasurface. (**A**) Calculated delay-dependent intensities of the SH waves with H- and V-polarization, where τ_0_ = 221.3 fs and the wavelength of 1220 nm of the pump wave are used for calculation. (**B**) Experimental setup for the ultrafast all-optical polarization switching. λ/2, half-wave plate; PBS, polarization beam splitter; M, mirror; DL, delay line; L, lens; S, sample; OL, objective lens; F, short-pass filter; P, linear polarizer; FM, flip mirror; SM, spectrometer. (**C**) Measured delay-dependent V-polarized SHG intensity from the uniform plasmonic metasurface. The delay-dependent SHG intensity is fitted by the Gaussian function (solid line) with a full-width at half-maximum (FWHM) of 521 fs. sCMOS, scientific complementary metal-oxide semiconductor.

As is shown in Fig. 3A, the H-polarized SH wave exhibits a Gaussian envelope with oscillating signal, and the V-polarized one shows a simple Gaussian curve. The width of the V-polarized SHG signal is dependent on the pulse width of the pump wave, indicating the pulse-width limited all-optical switching. For the proposed all-optical ultrafast switch, the full width at half-maximum (FWHM) pulse width of the pumping wave ( $2\sqrt{\text{ln}2}\tau_{0}$ ) will determine the shortest switching time. Moreover, the SHG signal is close to zero at the delay time much longer than the pulse width, which means that the optical switching is background-free, and the theoretical modulation depth is up to 100%.

The experimental setup used for verifying the all-optical ultrafast polarization switching of the SH waves with the uniform plasmonic metasurface is shown in Fig. 3B. A kilohertz femtosecond laser pumped optical parametric amplifier (OPA) was used as the pump laser. The pump wavelength was set to be 1220 nm, where the plasmonic metasurface shows the strongest SHG response. Then, the pump wave was split into two beams with H and V polarization. A variable and fixed time delays are introduced into the H- and V-polarized pump beams. The two pump beams were collinearly focused onto the plasmonic metasurface, and the SH waves were generated. The V-polarization components of the SH waves were measured by using a spectrometer. The measured delay-dependent V-polarized SHG intensity is shown in Fig. 3C and fitted by a Gaussian function. The FWHM of the fitted Gaussian curve is $2\sqrt{2}\sqrt{\text{ln}2}\tau_{0}$ ~ 521 fs, from which we can extract the pulse width of the OPA laser pulse $2\sqrt{\text{ln}2}\tau_{0}$ ~ 369 fs. It can be found that the switching time becomes longer as the pulse width increases. Such a short switching time indicates the potential of all-optical ultrafast polarization switching with terahertz speed. The modulation depth of the metasurface optical switch is approximately 97%, which is close to the theoretical limit.

**Fig. 4.**
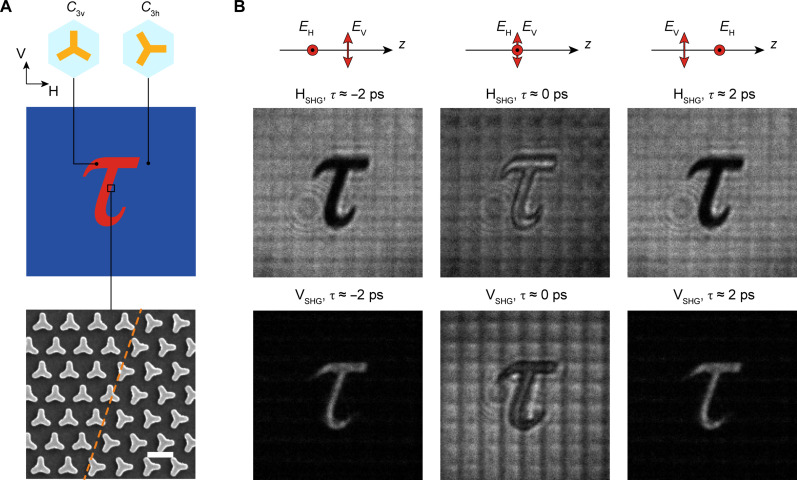
All-optical ultrafast polarization switching with the dual-channel plasmonic metasurface. (**A**) Schematic diagram of the dual-channel plasmonic metasurface with a τ pattern. In the region of τ (red), the meta-atoms are *C*_3v_ (one arm vertical), and the surrounding meta-atoms are *C*_3h_ (one arm horizontal). The SEM image of the metasurface is shown below (scale bar, 500 nm), in which the orange dashed line marks the boundary between the two kinds of meta-atoms. (**B**) Captured SHG images from the metasurface for the two pump waves with H- and V-polarization and different delay time (τ).

To further demonstrate the capability of parallel information processing of the all-optical ultrafast switch, we designed and fabricated the dual-channel plasmonic metasurface, which is composed of pattern-determined meta-atoms with different orientations. As shown in Fig. 4A, the meta-atoms in the “τ” pattern region are *C*_3h_ (one arm horizontal) and the outside are *C*_3v_ (one arm vertical). The SHG images from the patterned metasurface were captured with the experimental setup shown in Fig. 3B. The captured V- and H-polarized SH images at different delay times are shown in Fig. 4B. The sketches above each column show the relative delay of the pump waves with H- and V-polarization (*E*_H_ and *E*_V_). It can be seen that the τ region radiates V-polarized SH waves when the delay time is larger than the pulse width (the first and third columns of Fig. 4B). When the two pump waves coincide in the time domain (the middle column of Fig. 4B), the τ region emits H-polarized SH waves. In comparison, the surrounding area has a complementary SHG intensity distribution with respect to the τ region. The ultrafast polarization switching of a binary image demonstrates the capability of parallel information processing with the metasurface optical switch.

## DISCUSSION

In conclusion, we demonstrate the all-optical ultrafast polarization switching of second-harmonic waves with the nonlinear plasmonic metasurfaces working in the near-infrared regime. By using the concept of geometric phase, the linear polarization directions of the second-harmonic waves could be well controlled by the linear polarization direction and time delay of the two pump waves. A switching time of 521 fs and a modulation depth of 97% were experimentally achieved. In addition, we also demonstrate the dual-channel all-optical ultrafast switching by designing the distributions of the meta-atoms. The metasurface platform is easy to be scaled up and allows for the design of parallel switching channels. To realize an all-optical ultrafast switch for practical application, we may need a kind of photorefractive material in which the temporal delay of the pump wave can be simultaneously controlled by a strong laser beam which is synchronized to it in the time domain. This work may provide a promising route for all-optical ultrafast information processing with nonlinear photonic metasurfaces. The proposed methodology can be used to control the vectorial fields, spin and orbital angular momentum of light in the time domain. A lot of functions such as all-optical logic gating, optical auto-correlation, and time-varying optical holography can be developed.

## MATERIALS AND METHODS

### Fabrication of plasmonic metasurfaces

The uniform and dual-channel plasmonic metasurfaces were fabricated by using standard electron beam lithography (EBL) and metal lift-off process as described below. A 0.7-mm-thick glass with a 15-nm-thick ITO film was chosen as the substrate. A 128-nm-thick positive electron resist (PMMA 950) was spin-coated onto the ITO glass and then baked at temperature of 180°C for 3 min. The designed pattern of metasurface was transferred to the electron resist by the EBL process, after which the resist was developed. Afterward, a 30-nm-thick gold film was deposited onto the resist by electron beam evaporation technique. Last, the plasmonic metasurface was obtained after a lift-off process.

### Nonlinear optical properties of the uniform plasmonic metasurface

A femtosecond laser pumped OPO (Coherent Chameleon) was used as the laser source. The output wavelength was tuned from 1150 to 1550 nm. For the wavelength-dependent experiment, the pumping power was set to be the equal. A linear polarizer and a quarter-wave plate were used to control the circular polarization states of the pump wave. The pump wave was focused onto the metasurface sample by a 4× infinity-corrected plan achromatic objective lens (Olympus RMS4X). The SH was collected by a 10× infinity-corrected plan achromatic objective lens (Olympus RMS10X). Then, a short-pass filter (Thorlabs FESH0800) was used to block the pump wave. A spectrometer (Andor SR-500i) equipped with an EMCCD (electron-multiplying charge coupled device) detector was used for measuring the spectra of the SH waves.

### All-optical ultrafast polarization switching

The femtosecond laser (Coherent, Astrella, repetition rate: 1 kHz) pumped OPA (Coherent OPerA Solo) output at a wavelength of 1220 nm was used as the pump laser. The laser was split into two beams with H- and V-polarizations by a polarization beam splitter (PBS). The power of the two beams was equalized by adjusting a half-wave plate (λ/2). A variable time delay was introduced into the H-polarized beam by using a delay line (Newport DLS225). The two beams were collinearly combined with the second PBS and focused onto the metasurface sample with a lens (focal length: 400 mm). The transmitted pump waves and SH wave were collected by a 4× infinity-corrected plan achromatic objective lens (Olympus RMS4X). After that, the pump beams were blocked by the short pass filters. The polarization state of the SH wave is analyzed by using a linear polarizer. The flip mirror was moved out of the optical path for characterizing the spectra of the SH wave and moved in for capturing the SHG image of dual-channel metasurface with a scientific complementary metal-oxide semiconductor camera (Andor Neo 5.5). The spectra and power of the SH waves were measured by using the spectrometer (Princeton Instruments IsoPlane SCT320) equipped with an EMCCD detector (Princeton Instruments ProEM).

### Nonlinear optical calculations

To better understand the nonlinear optical properties of the gold-ITO hybrid metasurface, the COMSOL Multiphysics program based on hydrodynamic model (*50*) was used to calculate the wavelength-dependent SHG responsivity (section S3). In the nonlinear optical calculations, the refractive index of gold is taken from the experimental results in the literature (*51*); the refractive index of the ITO film was measured by using a spectroscopic ellipsometer, and the ENZ wavelength is around 1160 nm (*46*). The nonlinear susceptibility of gold and ITO film is from the measured data in (*30*) and (*52*), respectively.

## Supplementary Materials

### This PDF file includes:

Section S1 to S3 Figs. S1 to S3

## References

[1] C. T. Phare, Y. H. Daniel Lee, J. Cardenas, M. Lipson, Graphene electro-optic modulator with 30 GHz bandwidth. Nat. Photonics 9, 511–514 (2015).

[2] C. Wang, M. Zhang, X. Chen, M. Bertrand, A. Shams-Ansari, S. Chandrasekhar, P. Winzer, M. Loncar, Integrated lithium niobate electro-optic modulators operating at CMOS-compatible voltages. Nature 562, 101–104 (2018). 30250251 10.1038/s41586-018-0551-y

[3] M. He, M. Xu, Y. Ren, J. Jian, Z. Ruan, Y. Xu, S. Gao, S. Sun, X. Wen, L. Zhou, L. Liu, C. Guo, H. Chen, S. Yu, L. Liu, X. Cai, High-performance hybrid silicon and lithium niobate Mach–Zehnder modulators for 100 Gbit s^−1^ and beyond. Nat. Photonics 13, 359–364 (2019).

[4] M. Li, J. Ling, Y. He, U. A. Javid, S. Xue, Q. Lin, Lithium niobate photonic-crystal electro-optic modulator. Nat. Commun. 11, 4123 (2020). 32807775 10.1038/s41467-020-17950-7 PMC7431411

[5] N. Savage, Acousto-optic devices. Nat. Photonics 4, 728–729 (2010).

[6] S. Ohkoshi, S. Takano, K. Imoto, M. Yoshikiyo, A. Namai, H. Tokoro, 90-degree optical switching of output second-harmonic light in chiral photomagnet. Nat. Photonics 8, 65–71 (2014).

[7] L. A. Williamson, Y. H. Chen, J. J. Longdell, Magneto-optic modulator with unit quantum efficiency. Phys. Rev. Lett. 113, 203601 (2014). 25432041 10.1103/PhysRevLett.113.203601

[8] P. Zhang, T. F. Chung, Q. Li, S. Wang, Q. Wang, W. L. B. Huey, S. Yang, J. E. Goldberger, J. Yao, X. Zhang, All-optical switching of magnetization in atomically thin CrI_3_. Nat. Mater. 21, 1373–1378 (2022). 36109674 10.1038/s41563-022-01354-7

[9] N. S. Patel, K. L. Hall, K. A. Rauschenbach, Interferometric all-optical switches for ultrafast signal processing. Appl. Optics 37, 2831–2842 (1998). 10.1364/ao.37.002831 18273229

[10] V. R. Almeida, C. A. Barrios, R. R. Panepucci, M. Lipson, All-optical control of light on a silicon chip. Nature 431, 1081–1084 (2004). 15510144 10.1038/nature02921

[11] X. Hu, P. Jiang, C. Ding, H. Yang, Q. Gong, Picosecond and low-power all-optical switching based on an organic photonic-bandgap microcavity. Nat. Photonics 2, 185–189 (2008).

[12] K. Nozaki, T. Tanabe, A. Shinya, S. Matsuo, T. Sato, H. Taniyama, M. Notomi, Sub-femtojoule all-optical switching using a photonic-crystal nanocavity. Nat. Photonics 4, 477–483 (2010).

[13] T. Volz, A. Reinhard, M. Winger, A. Badolato, K. J. Hennessy, E. L. Hu, A. Imamoğlu, Ultrafast all-optical switching by single photons. Nat. Photonics 6, 605–609 (2012).

[14] K. F. MacDonald, Z. L. Sámson, M. I. Stockman, N. I. Zheludev, Ultrafast active plasmonics. Nat. Photonics 3, 55–58 (2009).

[15] T. Utikal, M. I. Stockman, A. P. Heberle, M. Lippitz, H. Giessen, All-optical control of the ultrafast dynamics of a hybrid plasmonic system. Phys. Rev. Lett. 104, 113903 (2010). 20366478 10.1103/PhysRevLett.104.113903

[16] M. Abb, Y. Wang, C. H. de Groot, O. L. Muskens, Hotspot-mediated ultrafast nonlinear control of multifrequency plasmonic nanoantennas. Nat. Commun. 5, 4869 (2014). 25189713 10.1038/ncomms5869

[17] M. R. Shcherbakov, P. P. Vabishchevich, A. S. Shorokhov, K. E. Chong, D. Y. Choi, I. Staude, A. E. Miroshnichenko, D. N. Neshev, A. A. Fedyanin, Y. S. Kivshar, Ultrafast all-optical switching with magnetic resonances in nonlinear dielectric nanostructures. Nano Lett. 15, 6985–6990 (2015). 26393983 10.1021/acs.nanolett.5b02989

[18] L. H. Nicholls, F. J. Rodriguez-Fortuno, M. E. Nasir, R. M. Cordova-Castro, N. Olivier, G. A. Wurtz, A. V. Zayats, Ultrafast synthesis and switching of light polarization in nonlinear anisotropic metamaterials. Nat. Photonics 11, 628–633 (2017).

[19] M. Taghinejad, H. Taghinejad, Z. Xu, Y. Liu, S. P. Rodrigues, K. T. Lee, T. Lian, A. Adibi, W. Cai, Hot-electron-assisted femtosecond all-optical modulation in plasmonics. Adv. Mater. 30, 1704915 (2018). 10.1002/adma.201704915 29333735

[20] M. Taghinejad, H. Taghinejad, Z. Xu, K. T. Lee, S. P. Rodrigues, J. Yan, A. Adibi, T. Lian, W. Cai, Ultrafast control of phase and polarization of light expedited by hot-electron transfer. Nano Lett. 18, 5544–5551 (2018). 30071164 10.1021/acs.nanolett.8b01946

[21] K. Wang, M. Li, H. H. Hsiao, F. Zhang, M. Seidel, A. Y. Liu, J. Chen, E. Devaux, C. Genet, T. Ebbesen, High contrast, femtosecond light polarization manipulation in epsilon-near-zero material coupled to a plasmonic nanoantenna array. ACS Photonics 8, 2791–2799 (2021).

[22] K. Wang, A. Y. Liu, H. H. Hsiao, C. Genet, T. Ebbesen, Large optical nonlinearity of dielectric nanocavity-assisted Mie resonances strongly coupled to an epsilon-near-zero mode. Nano Lett. 22, 702–709 (2022). 34994573 10.1021/acs.nanolett.1c03876

[23] Y. M. Yang, K. Kelley, E. Sachet, S. Campione, T. S. Luk, J. P. Maria, M. B. Sinclair, I. Brener, Femtosecond optical polarization switching using a cadmium oxide-based perfect absorber. Nat. Photonics 11, 390–395 (2017).

[24] Q. Guo, R. Sekine, L. Ledezma, R. Nehra, D. J. Dean, A. Roy, R. M. Gray, S. Jahani, A. Marandi, Femtojoule femtosecond all-optical switching in lithium niobate nanophotonics. Nat. Photonics 16, 625–631 (2022).

[25] P. J. Guo, R. D. Schaller, J. B. Ketterson, R. P. H. Chang, Ultrafast switching of tunable infrared plasmons in indium tin oxide nanorod arrays with large absolute amplitude. Nat. Photonics 10, 267–273 (2016).

[26] M. Z. Alam, I. De Leon, R. W. Boyd, Large optical nonlinearity of indium tin oxide in its epsilon-near-zero region. Science 352, 795–797 (2016). 27127238 10.1126/science.aae0330

[27] M. Z. Alam, S. A. Schulz, J. Upham, I. De Leon, R. W. Boyd, Large optical nonlinearity of nanoantennas coupled to an epsilon-near-zero material. Nat. Photonics 12, 79–83 (2018).

[28] H. Wang, L. Sun, K. Du, W. Zhang, S. J. Chua, G. Li, T. Mei, Thermal energy dependent transient permittivity of epsilon-near-zero material. Sci. China Phys. Mech. Astron. 65, 284211 (2022).

[29] M. Ono, M. Hata, M. Tsunekawa, K. Nozaki, H. Sumikura, H. Chiba, M. Notomi, Ultrafast and energy-efficient all-optical switching with graphene-loaded deep-subwavelength plasmonic waveguides. Nat. Photonics 14, 37–43 (2020).

[30] F. X. Wang, F. J. Rodríguez, W. M. Albers, R. Ahorinta, J. E. Sipe, M. Kauranen, Surface and bulk contributions to the second-order nonlinear optical response of a gold film. Phys. Rev. B 80, 233402 (2009).

[31] Y. R. Shen, *The Principles of Nonlinear Optics* (John Wiley & Sons, 2002).

[32] R. W. Boyd, *Nonlinear optics* (Academic, San Diego, 2019).

[33] X. Yin, Z. Ye, D. A. Chenet, Y. Ye, K. O’Brien, J. C. Hone, X. Zhang, Edge nonlinear optics on a MoS2Atomic monolayer. Science 344, 488–490 (2014). 24786072 10.1126/science.1250564

[34] K. L. Seyler, J. R. Schaibley, P. Gong, P. Rivera, A. M. Jones, S. Wu, J. Yan, D. G. Mandrus, W. Yao, X. Xu, Electrical control of second-harmonic generation in a WSe_2_ monolayer transistor. Nat. Nanotechnol. 10, 407–411 (2015). 25895004 10.1038/nnano.2015.73

[35] T. Zentgraf, A. Christ, J. Kuhl, H. Giessen, Tailoring the ultrafast dephasing of quasiparticles in metallic photonic crystals. Phys. Rev. Lett. 93, 243901 (2004). 15697811 10.1103/PhysRevLett.93.243901

[36] M. Taghinejad, Z. Xu, K. T. Lee, T. Lian, W. Cai, Transient second-order nonlinear media: Breaking the spatial symmetry in the time domain via hot-electron transfer. Phys. Rev. Lett. 124, 013901 (2020). 31976680 10.1103/PhysRevLett.124.013901

[37] J. Y. Shan, M. Ye, H. Chu, S. Lee, J. G. Park, L. Balents, D. Hsieh, Giant modulation of optical nonlinearity by Floquet engineering. Nature 600, 235–239 (2021). 34880426 10.1038/s41586-021-04051-8

[38] N. Sirica, P. P. Orth, M. S. Scheurer, Y. M. Dai, M. C. Lee, P. Padmanabhan, L. T. Mix, S. W. Teitelbaum, M. Trigo, L. X. Zhao, G. F. Chen, B. Xu, R. Yang, B. Shen, C. Hu, C. C. Lee, H. Lin, T. A. Cochran, S. A. Trugman, J. X. Zhu, M. Z. Hasan, N. Ni, X. G. Qiu, A. J. Taylor, D. A. Yarotski, R. P. Prasankumar, Photocurrent-driven transient symmetry breaking in the Weyl semimetal TaAs. Nat. Mater. 21, 62–66 (2022). 34750539 10.1038/s41563-021-01126-9

[39] S. Klimmer, O. Ghaebi, Z. Y. Gan, A. George, A. Turchanin, G. Cerullo, G. Soavi, All-optical polarization and amplitude modulation of second-harmonic generation in atomically thin semiconductors. Nat. Photonics 15, 837–842 (2021).

[40] Y. Zhang, X. Bai, J. Arias Munoz, Y. Dai, S. Das, Y. Wang, Z. Sun, Coherent modulation of chiral nonlinear optics with crystal symmetry. Light Sci. Appl. 11, 216 (2022). 35803908 10.1038/s41377-022-00915-4 PMC9270472

[41] Y. Zhang, Y. Wang, Y. Dai, X. Bai, X. Hu, L. Du, H. Hu, X. Yang, D. Li, Q. Dai, T. Hasan, Z. Sun, Chirality logic gates. Sci. Adv. 8, eabq8246 (2022). 36490340 10.1126/sciadv.abq8246 PMC9733934

[42] M. W. Klein, C. Enkrich, M. Wegener, S. Linden, Second-harmonic generation from magnetic metamaterials. Science 313, 502–504 (2006). 16873661 10.1126/science.1129198

[43] K. Konishi, T. Higuchi, J. Li, J. Larsson, S. Ishii, M. Kuwata-Gonokami, Polarization-controlled circular second-harmonic generation from metal hole arrays with threefold rotational symmetry. Phys. Rev. Lett. 112, 135502 (2014). 24745436 10.1103/PhysRevLett.112.135502

[44] N. Segal, S. Keren-Zur, N. Hendler, T. Ellenbogen, Controlling light with metamaterial-based nonlinear photonic crystals. Nat. Photonics 9, 180–184 (2015).

[45] C. McDonnell, J. Deng, S. Sideris, T. Ellenbogen, G. Li, Functional THz emitters based on Pancharatnam-Berry phase nonlinear metasurfaces. Nat. Commun. 12, 30 (2021). 33397951 10.1038/s41467-020-20283-0 PMC7782718

[46] J. Deng, Y. Tang, S. Chen, K. Li, A. V. Zayats, G. Li, Giant enhancement of second-order nonlinearity of Epsilon-near- Zero medium by a plasmonic metasurface. Nano Lett. 20, 5421–5427 (2020). 32496801 10.1021/acs.nanolett.0c01810

[47] G. Li, S. Chen, N. Pholchai, B. Reineke, P. W. Wong, E. Y. Pun, K. W. Cheah, T. Zentgraf, S. Zhang, Continuous control of the nonlinearity phase for harmonic generations. Nat. Mater. 14, 607–612 (2015). 25849530 10.1038/nmat4267

[48] G. Li, S. Zhang, T. Zentgraf, Nonlinear photonic metasurfaces. Nat. Rev. Mater. 2, 17010 (2017).

[49] A. Karnieli, Y. Li, A. Arie, The geometric phase in nonlinear frequency conversion. Front. Phys. 17, 12301 (2021).

[50] C. Ciraci, E. Poutrina, M. Scalora, D. R. Smith, Origin of second-harmonic generation enhancement in optical split-ring resonators. Phys. Rev. B 85, 201403 (2012).

[51] P. B. Johnson, R. W. Christy, Optical constants of the noble metals. Phys. Rev. B 6, 4370–4379 (1972).

[52] A. Capretti, Y. Wang, N. Engheta, L. Dal Negro, Comparative study of second-harmonic generation from epsilon-near-zero indium tin oxide and titanium nitride nanolayers excited in the near-infrared spectral range. ACS Photonics 2, 1584–1591 (2015).

