# Supplementary material for: All-optical ultrafast polarization switching with nonlinear plasmonic metasurfaces
Source: Sci Adv. 2024 Feb 21;10(8):eadk3882. doi: 10.1126/sciadv.adk3882 (PMC10881032; doi:10.1126/sciadv.adk3882)
Supplement: Supplementary file 1 — Section S1 to S3 Figs. S1 to S3 [file sciadv.adk3882_sm.pdf]

Science Advances
AAAS

Supplementary Materials for

All-optical ultrafast polarization switching with nonlinear plasmonic metasurfaces

Heng Wang *et al.*

Corresponding author: Guixin Li, ligx@sustech.edu.cn

Sci. Adv. **10**, eadk3882 (2024)
DOI: 10.1126/sciadv.adk3882

This PDF file includes:

Sections S1 to S3
Figs. S1 to S3

Section 1. Working principle of the all-optical ultrafast polarization switching

From the fundamentals of nonlinear optics (*31, 32*), we know that the second-order nonlinear polarization can be represented as

$$P_i^{(2\omega)} = \varepsilon_0 \sum_{jk} \chi_{ijk}^{(2)} E_j^{(\omega)} E_k^{(\omega)} , \tag{S1}$$

where ε_0 is the vacuum permittivity, $\chi_{ijk}^{(2)}$ is the second-order susceptibility tensor, and the indices (i, j, k) run over the Cartesian coordinates (x, y, z). The nonvanishing elements of the second-order susceptibility tensor for the C_{3h} meta-atom (one arm horizontal) are

$$\chi_{xxx}^{(2)} = -\chi_{xyy}^{(2)} = -\chi_{yxy}^{(2)} = -\chi_{yyx}^{(2)} , \tag{S2}$$

and for the C_{3v} meta-atom (one arm vertical), the nonvanishing elements are

$$\chi_{yyy}^{(2)} = -\chi_{yxx}^{(2)} = -\chi_{xyx}^{(2)} = -\chi_{xxy}^{(2)} . \tag{S3}$$

Under contracted notation, the nonlinear susceptibility tensor could be represented in the matrix form. Assuming the pump laser in normally incident on the C_{3h} plasmonic meta-atom along its three-fold rotational axis, the nonlinear polarization for a SHG process can be written as

$$\begin{bmatrix} P_x^{(2\omega)} \\ P_y^{(2\omega)} \\ P_z^{(2\omega)} \end{bmatrix} = 2\varepsilon_0 \begin{bmatrix} d_{11} & d_{12} & 0 & 0 & 0 & 0 \\ 0 & 0 & 0 & 0 & 0 & d_{26} \\ 0 & 0 & 0 & 0 & 0 & 0 \end{bmatrix} \begin{bmatrix} \left(E_x^{(\omega)}\right)^2 \\ \left(E_y^{(\omega)}\right)^2 \\ \left(E_z^{(\omega)}\right)^2 \\ 2E_y^{(\omega)} E_z^{(\omega)} \\ 2E_x^{(\omega)} E_z^{(\omega)} \\ 2E_x^{(\omega)} E_y^{(\omega)} \end{bmatrix} , \tag{S4}$$

where $d_{11} = -d_{12} = -d_{26}$. Then, we can obtain the nonlinear polarization as

$$\begin{bmatrix} P_x^{(2\omega)} \\ P_y^{(2\omega)} \\ P_z^{(2\omega)} \end{bmatrix} \propto \begin{bmatrix} \left(E_x^{(\omega)}\right)^2 - \left(E_y^{(\omega)}\right)^2 \\ -2E_x^{(\omega)} E_y^{(\omega)} \\ 0 \end{bmatrix} . \tag{S5}$$

In the meantime, the time-domain electric fields of the pump wave with linear polarizations along x (H) and y (V) directions are

$$\begin{cases} E_x^{(\omega)} = A_1(t+\tau)\exp\left[-i\omega(t+\tau)\right] \\ E_y^{(\omega)} = A_2(t)\exp(-i\omega t) \\ E_z^{(\omega)} = 0 \end{cases}, \tag{S6}$$

Substituting Equation (S6) into Equation (S5), we obtain the time-dependent nonlinear polarization of the SH waves

$$\begin{bmatrix} P_x^{(2\omega)} \\ P_y^{(2\omega)} \\ P_z^{(2\omega)} \end{bmatrix} \propto \begin{bmatrix} A_1^2(t+\tau)\exp\left[-2i\omega(t+\tau)\right] - A_2^2(t)\exp(-2i\omega t) \\ -2A_1(t+\tau)A_2(t)\exp\left[-i\omega(2t+\tau)\right] \\ 0 \end{bmatrix}, \tag{S7}$$

Then, the τ-dependent SHG intensity could be further calculated by integrating the square of the amplitudes of the electric fields of the SH waves

$$\begin{bmatrix} I_x^{(2\omega)} \\ I_y^{(2\omega)} \end{bmatrix} \propto \begin{bmatrix} \int_{-\infty}^{+\infty} A_1^4(t+\tau)\,dt + \int_{-\infty}^{+\infty} A_2^4(t)\,dt - 2\cos(2\omega\tau)\int_{-\infty}^{+\infty} A_1^2(t+\tau)A_2^2(t)\,dt \\ 4\int_{-\infty}^{+\infty} A_1^2(t+\tau)A_2^2(t)\,dt \end{bmatrix}. \tag{S8}$$

Assuming the electric field amplitudes of the two pump waves are equal and the pulse shapes in the time domain are same as each other, i.e. $A_1 = A_2 = A$. For a Gaussian pulse, the envelope function of the electric field in time domain could be written as $A(t) = \exp\left[-t^2/\left(2\tau_0^2\right)\right]$, where τ_0 is a parameter that determines the pulse width. Note that the full-width at half-maximum (FWHM) of the pulse intensity for the pump waves is $2\sqrt{\ln 2}\times\tau_0$. Substituting the Gaussian function of $A(t)$ into Equation (S8), the τ-dependent SHG power is derived as

$$\begin{bmatrix} I_x^{(2\omega)} \\ I_y^{(2\omega)} \end{bmatrix} \propto \begin{bmatrix} 1 - \cos(2\omega\tau)\exp\left[-\tau^2/\left(2\tau_0^2\right)\right] \\ 2\exp\left[-\tau^2/\left(2\tau_0^2\right)\right] \end{bmatrix}. \tag{S9}$$

Section 2. Linear and nonlinear optical properties of the uniform plasmonic metasurface

A broadband continuous-wave lamp (Alltion XD-301-150W) was used as the light source of the linear transmission experimental setup, and the spectra were measured by visible and near-infrared spectrometers (Ocean Optics USB2000+ and Flame-NIR). The linear transmission spectra of the uniform plasmonic metasurface (Fig. 2A) were measured with the four polarization combinations of the incident and transmitted light, as shown in Fig. S1. It can be seen that there is a broad transmission valley in the wavelength range between 1100 nm and 1500 nm. From our previous studies (*46*), we know that this valley corresponds to the localized plasmonic resonance of the meta-atoms. Together with the epsilon-near-zero effect of the ITO layer at wavelength ~ 1200 nm, the gold plasmonic metasurface show strong SHG responses for the pump wavelength between 1150 nm and 1350 nm.

The properties of the SH waves from the uniform plasmonic metasurface were firstly characterized by using the OPO system (Materials and Methods). At pump wavelength of 1220 nm where SHG has the strongest responses (Fig. 2), the circularly polarized pump wave is normally incident on the metasurface. Then, four polarization combinations were used to characterize the properties of the SHG spectra (Fig. S2A). The results agree well with the symmetry selection rules in nonlinear optics (*43, 48*). Figure S2B shows the power-dependent SHG intensities for the LCP_{pump}–RCP_{SH} and RCP_{pump}–LCP_{SH} measurement schemes. The fitted slope values are 1.96 and 1.95, respectively, which agree well with the theoretical value of 2.0 for the SHG process. The SHG power is ~ 1.4 pW for the pumping power of ~ 3.54 mW.

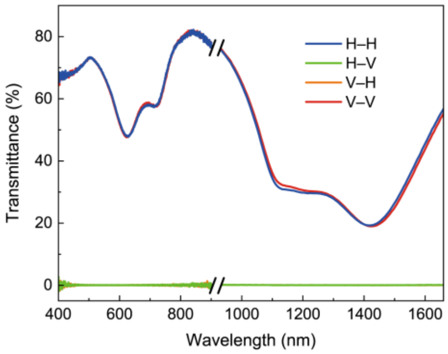

Fig. S1. **Measured polarization-resolved transmission spectra of the uniform plasmonic metasurface.** H, Horizontal polarization; V, Vertical polarization. H-H, H-V, V-H and V-V are the four polarization combinations in the measurements. The first and second character represent the linear polarization states of the incident and transmitted light, respectively.

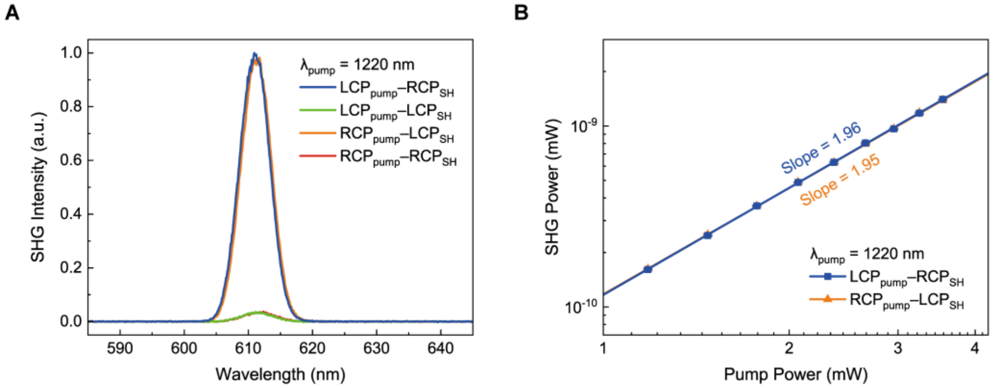

Fig. S2. Measured nonlinear optical properties of the uniform plasmonic metasurface. The metasurface was excited by a femtosecond laser from the OPO system. The pump wavelength is 1220 nm. (**A**) Four circular polarization combinations were used to characterize the properties of the SH waves. (**B**) Measured power-dependent SHG intensities for the LCP_{pump}–RCP_{SH} and RCP_{pump}–LCP_{SH} measurement schemes.

Section 3. Calculated nonlinear optical properties of the gold-ITO hybrid metasurface

To qualitatively match the experimental results in Fig. 2 of main text, the arm length, width and thickness of the meta-atom are chosen to be 200 nm, 80 nm and 30 nm, respectively in the calculation (Fig. S3A). The meta-atoms are arranged in a hexagonal lattice with a period of 550 nm. The calculated polarization-resolved transmission spectra of the metasurface are shown in Fig. S3B, which agree well with the measured ones shown in Fig. S1. The calculated polarization-resolved SHG responses from the metasurface for linearly polarized pump wave are shown in Fig. S3C. The surface nonlinear susceptibilities of gold are $\chi^{(2)}_{\mathrm{nnn}} = 442\ \mathrm{pm^2/V}$, $\chi^{(2)}_{\mathrm{ntt}} = 4940\ \mathrm{pm^2/V}$ and $\chi^{(2)}_{\mathrm{ttn}} = 1770\ \mathrm{pm^2/V}$ (*30*); the bulk nonlinear susceptibility of ITO is $\chi^{(2)}_{zzz} = 0.18\ \mathrm{pm/V}$ (*52*). From either horizontally or vertically polarized pump wave (H_{pump} or V_{pump}), the generated second-harmonic waves are mainly horizontally polarized (H_{SH}), and the vertical components are negligible. These polarization properties agree well with the experimental results in Fig. 2D. If the nonlinear susceptibility of ITO is set to be zero (Fig. S3D), we found that the SHG efficiency of the gold-ITO hybrid metasurface is ~ 60 times lower than that of the case in Fig. S3C. Thus, we can conclude that the ITO layer plays important roles in improving SHG efficiency of the hybrid metasurface.

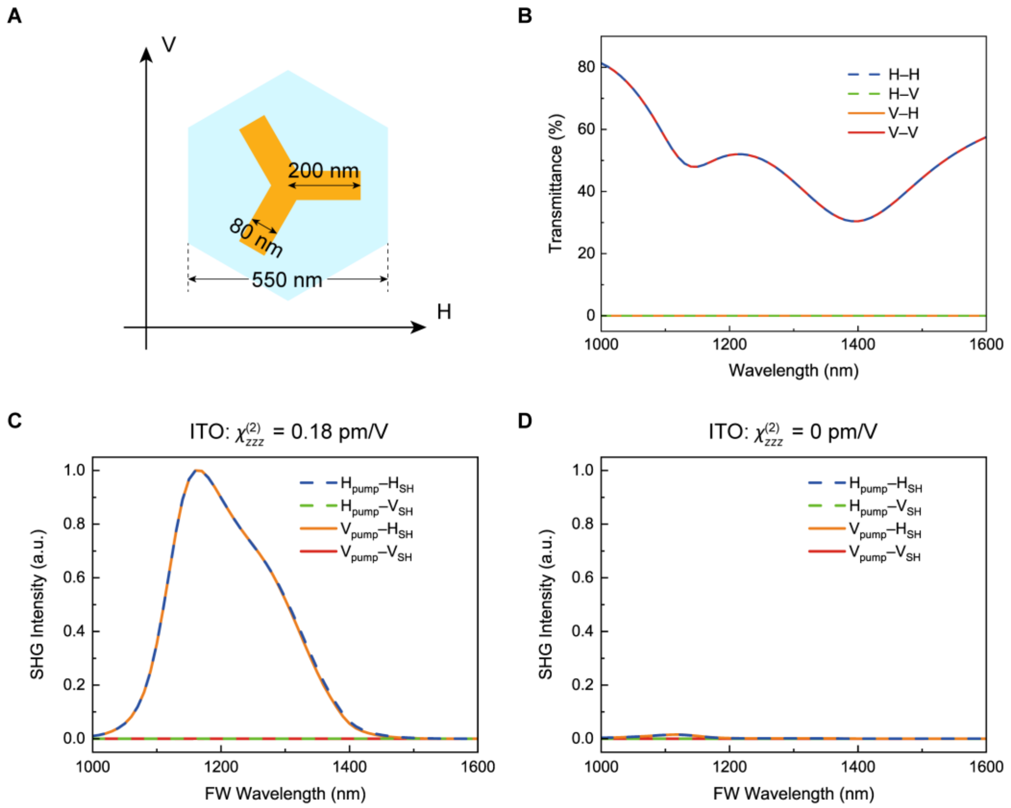

Fig. S3. The calculated linear and nonlinear optical properties of the gold-ITO hybrid metasurface. (**A**) The geometrical parameters of a C_{3h} gold meta-atom. The arm length, width and thickness of the meta-atom are 200 nm, 80 nm and 30 nm, respectively. (**B**) Calculated polarization-resolved transmission spectra of the gold-ITO hybrid plasmonic metasurface. (**C**) and (**D**) Calculated polarization-resolved SHG responses of the gold-ITO hybrid metasurface. H_{pump} and V_{pump} represent the horizontally and vertically polarized pump waves; H_{SH} and V_{SH} correspond to horizontal and vertical polarizations of the second harmonic waves. The nonlinear susceptibility of ITO is set as 0.18 pm/V (C) and 0 pm/V (D), respectively.
